# Cost of care for asylum seekers and refugees entering the United States: The case of volunteer medical providers in El Paso, Texas

**DOI:** 10.1371/journal.pone.0278386

**Published:** 2022-12-01

**Authors:** Rigoberto I. Delgado, Manuel De la Rosa, Marlon A. Picado, Lisa Ayoub-Rodriguez, Celia E. Gonzalez, Leopold Gemoets

**Affiliations:** 1 School of Biomedical Informatics, The University of Texas Health Science Center at Houston, Houston, Texas, United States of America; 2 Texas Tech University Health Sciences Center El Paso, El Paso, Texas, United States of America; 3 Department of Accounting and Information Systems, The University of Texas at El Paso, El Paso, Texas, United States of America; University of Georgia, UNITED STATES

## Abstract

**Background:**

Between October 2018, and February 2020, the United States saw an unprecedented increase in the number of asylum seekers and refugees arriving unexpectedly at international crossings along the US-Mexico Border. Many of these migrants needed proper medical attention, and consequently created significant pressure on local health systems. In El Paso, Texas, volunteer clinicians, collaborating closely with religious organizations and non-governmental organizations, provided outpatient medical care for the new arrivals; the county hospital provided in-patient care at local tax payers’ expense. The objective of this study was to estimate costs of healthcare services offered by these volunteers in order to formulate sustainable and appropriate healthcare policies to address the needs of refugees and asylum seekers in the United States.

**Methods:**

A mixed methods approach was used including personal interviews with stakeholders, and follow up surveys with volunteer clinicians. The cost analysis was done from the payer perspective using Medicaid reimbursement rates.

**Results:**

Total costs of care provided to asylum seekers and refugees varied between $1.9MM to $4.4MM during the study period. The number of patient visits was estimated at 15,736 to 19,236, and cost per patient ranged between $99 and $281. Most common conditions treated by volunteer providers were abdominal pain, dermatological conditions, headaches, dehydration and hypertension.

**Conclusions:**

This is the first study looking at the cost of healthcare for refugees and asylum seekers provided by volunteer clinicians, in a binational context. The resources invested by volunteer providers were significant, and essential to meet medical needs of migrant populations. Without appropriate financial support, a strategy relying on volunteer and local community resources will prove unsustainable in the long term. Findings from this study will help formulate federal and local policies to support local health systems along the US-Mexico Border in providing care to future migrations into the United States.

## Introduction

In the last quarter of 2018, the United States saw an unprecedented increase in the number of asylum seekers and refugees arriving unexpectedly at international crossings of Texas, New Mexico, Arizona and California. In fiscal year 2019 the number of unauthorized entrants taken into custody along the US-Mexico Border ascended to over 852,000 [1], which was more than double the numbers seen in 2018. In the month of May 2019 alone, there were over 144,000 people detained at the Border which included unaccompanied children, single adults, and family units. The nationality of entrants seen were different from previous waves as well, originating as far away as Central American and Brazil [2]. Many came bearing the strain of undiagnosed illnesses causing a disproportionate need for proper medical attention, and consequently creating significant pressure on local health systems.

In the case of El Paso, Texas, a city located along the US-Mexico Border, volunteer clinicians provided outpatient medical care for refugees, while critical care was provided at the County hospital at the expense of local taxpayers. A number of medical providers, collaborating closely with religious organizations and non-governmental organizations (NGOs), quickly arranged volunteer schedules to help treat infections, dehydrations, fractures and many other conditions. Volunteer doctors, nurses, and medical students worked evenings and weekends caring for a growing number of patients, while an overwhelmed immigration system tried to keep up with new arrivals [3, 4]. Without these clinician volunteers and community support organizations, an even larger humanitarian crisis would have resulted at the doorstep of the United States. The policies of the Trump Administration, which directed new arrivals to Mexico through the Migration Protection Protocols, or the “Remain in Mexico” Program, placed a hold on new entrants at the Border, shifting the enormity of the challenges and local resource needs to Mexico. With the Biden Administration’s reversal of the Trump policies [5–7], new waves of asylum seekers already started making their way North in hopes of leaving behind a life of social unrest [8]. These policies were short lived, however, and soon new arrivals were sent to Mexico to wait for a judicial decision, or repatriated to countries such as Haiti [9]. Yet the number of migrants seeking to relocate to the US continues acerbated by economic hardship, political instability, and conditions with the COVID-19 pandemic at their home countries.

This study presents estimates of the cost of providing healthcare services to asylum seekers and refugees arriving in El Paso during the 2018–2020 period. The goal is to provide baseline data that can be used for formulating appropriate federal and local policies to support local health systems along the US-Mexico Border, and avoid a repetition of the migrant chaos of 2018–2020. Currently, no research has been done on the costs of care for migrants. Also, this is the first study looking at the cost of healthcare for refugees and asylum seekers provided by volunteer clinicians, in the context of the US-Mexico Border. We conclude with various observations and recommendations for policy regarding healthcare services for asylum seekers and refugees. In two follow up studies, we will present estimates of in-hospital costs of care for asylum seeker and refugees in El Paso, as well as medical treatment costs for those individuals relocated to the neighboring city of Juarez, Mexico. This is to give estimates of costs of care from a regional perspective, which includes a common binational area of the United Stated and Mexico. We believe that only by considering costs of care for migrant populations on both sides of the US-Mexico Border is an essential first step for sound policy planning. Exodus of large numbers of people are common in other countries particularly in Europe, and findings from this study could also be useful for communities outside the United States.

Studies on the costs of care for migrant populations are limited, and relatively old. Furthermore, there is no known research involving costs of voluntary medical services such as the situation seen in El Paso during the 2018–2020 period. Most studies are related to in-hospital care or preventive programs managed by established institutional resources in the United States or other countries. A study from Turkey, for example, estimated costs of emergency department resource use by Syrian refugees, concluding that over the span of one year (2015) total costs of emergency department visits amounted to over $774MM USD for the equivalent of almost 11,000 visits [10]. Other studies have focused on the costs of treating infectious diseases among migrant populations such as Chagas disease [11, 12]; the cost-effectiveness ratio of screening for Chagas among Latin America immigrants residing in a non-endemic area of Spain was estimated at 125€/QALY (Quality-Adjusted Life-Year) [13]. A study from Germany estimated average of costs of oral healthcare for refugees varying between 205.86 EUR (conservative treatment) and 588.0 EUR (prosthetic treatment) [14].

In the US several studies have focused on estimating cost savings of implementing vaccination programs for refugees prior to arrival into this country [15, 16], and costs of operating similar US domestic programs [17, 18]. A1999 study on screening and treating immigrants for intestinal parasites indicated an incremental cost-effectiveness ratio of $159,236 per DALY (disability-adjusted life-year) averted, and savings of $4.2 million per year in prevented deaths and hospitalizations [19]. A similar study estimated the cost-effectiveness of implementing strategies to diagnose and treat for latent tuberculosis infection (LTBI) acquired in a person’s country of origin [20]. A relatively new study using secondary data from 2012 [21], estimated the costs of controlling an outbreak of measles among refugees settled in Kentucky at $25,000.

Other studies have concentrated on estimating aggregate healthcare costs of migrant communities already residing in the US. A 2007 study by DuBard and Massing [22] showed that care for illegal US immigrants resulted in a 28% increase in emergency Medicaid spending in North Carolina over the 2001–2004 period. Pregnancy complications and childbirth were the most common procedures treated, but other conditions were common such as injuries, renal failure, gastrointestinal disease, and cardiovascular problems. Goldman, Smith, and Sood [23], estimated that in 2000 total healthcare resources consumed by the undocumented amounted to $6.4 billion, which was a significant small amount in proportion to expenditures incurred by the rest of the US population. A similar study concluded that overall total healthcare expenditures for immigrants (legal and illegal) residing in the United States was 55% lower than expenditures for US-born residents [24].

The issue faced by El Paso County authorities of providing care to migrants is not new. For several generations, Border communities have faced the need to support waves of arrivals with assistance of community organizations. Relying on volunteer providers to handle increasing numbers of refugees and asylum seekers is an unsustainable approach. Rather, a well-planned strategy leveraging local community and government resources is more likely to have a long-lasting impact. With the results from this study we expect to contribute evidence towards establishing such strategies.

## Methods

Our study focused on healthcare providers offering volunteer, non-compensated, care to refugees and asylum seekers entering the United States through El Paso, Texas, and detained by ICE during the period of October 2018 through February 15, 2020. A mixed methods methodology was used in the study, which included several approaches. First, an extensive review of existing records on provider and medical student voluntary hours collected by El Paso United Way for the period June 27, 2019, to February 15, 2020. The United Way of El Paso contributed resources during this period to assist in the coordination of volunteers. Second, and in order to estimate number of volunteer resources prior to May 1, 2019, we completed a series of in-depth-interviews with ten key informants who were involved in providing medical care from the start of the crisis on October, 2018. Coordination of volunteers during this period was mainly in charge of two of the authors of this study (De la Rosa and Ayoub-Rodriguez). Third, selected volunteer providers were interviewed in person (8), or with the use of an online survey (67). The survey included open-ended questions related to number of hours invested by volunteers, type of services provided (i.e. primary care, emergency care, and, prenatal care), conditions treated, and type of medications used.

Cost of medical personnel time was estimated using published reimbursement rates in Texas for Medicaid codes CPT- 99213 (Low to moderate severity problem, 15 min), and CPT-99214 (Moderate to high severity problem, 25 min), for 2018 and 2019, respectively. Among the volunteer providers were medical students, and their time was estimated considering volunteer hourly rates defined by the State of Texas at $25.10 per hour for 2018, and $25.47 per hour for 2019 [25]. Volunteer and Medicaid nominal rates used in the study were adjusted for inflation using the Consumer Price Index [26]. The cost analysis was done from the perspective of the payer, since costs estimates were based on Medicaid reimbursement rates. The perspective of analysis is an accepted definition in health economics [27–29], and indicates the type of costs considered in a specific study. The payer perspective, for example, takes into account only reimbursements to providers on the part of the health insurance payer and does not consider other costs fronted by stakeholders in an intervention (e.g., out-of-pocket costs to patients).

A sensitivity analysis was completed varying the number of patients treated, and reimbursement rate assumptions. The two Medicaid codes mentioned previously represented used to represent a possible reimbursement “floor”, or opportunity cost, of volunteer provider services. In addition, two additional reimbursement rate scenarios of 135% and 185% above Medicaid published average rates were included in the sensitivity analysis to model a “ceiling” of potential private insurance payments. The study did not include estimates of healthcare services to populations defined by the US Department of State as “Unaccompanied Minors” since these groups were not assigned to hospitality centers in the study. The study did include families and adults traveling alone. In addition, the data collected did not contain patient country of origin and socioeconomic conditions. The study received Institutional Review Board approval from the University of Texas at El Paso (IRBNet ID 1465096–3). Written participant consent was obtained from the interview and survey respondents.

## Results

Refugees and asylum seekers once reaching El Paso, Texas, were detained by either Immigration and Customs Enforcement (ICE), US Customs and Border Protection, or the Office of Refugee Resettlement. Once released by these agencies, refugees and asylum seekers were assigned to several hospitality centers to wait for their case hearing and possible resettlement, or deportation. Several hotels were contracted by hospitality centers, financed by ICE, to help accommodate the growing numbers of arriving refugees. Working mainly during weekday evenings and weekend days, volunteer providers at each of these centers would treat those arrivals needing care. Hospitality centers, stocked with donated medical supplies or purchased over-the-counter medications, had assigned areas to work as infirmaries. These were cramped areas where volunteer clinicians could administer medication and document a series of health-related issues with each patient. If patients needed additional care, they were transported to the county hospital, the University Medical Center of El Paso, or local private hospitals. The hospitality centers were operated mainly by local non-profit organizations, or facilities funded through private contributions, and religious institutions.

A total of 75 volunteer providers participated in the effort, and included physicians (55%), medical students (31%) nurses (8%), medical residents (4%), and physician assistants (3%). Table 1 shows the number of hours volunteer providers devoted per week, and type of care given to asylum seekers and refugees

**Table 1:** Average hours per week contributed by volunteer providers and type of care provided to care for asylum seekers and refugees entering El Paso, Texas (n = 75).

Percent of Providers by Number of Volunteer Hours per Week		Percent of Providers by Type of Care Provided*			
		Primary Care	Emergency Care	Prenatal care	Not specified
1–10 hrs.	33%	67%	33%	33%	-
10–20 hrs.	22%	-	-	-	100%
20–30 hrs.	33%	100%	100%	33%	-
Other	11%	-	-	-	11%
Total	100%	56%	44%	22%	33%

*Note: Percentages add to more than 100% since providers offered more than one type of care

A third (33%) of providers devoted between one and ten hours per week, while more than half (55%) dedicated between 10 to 30 hours per week– 33% dedicated up to 30 hrs. per week. Table 1 also indicates that up to 56% of providers were involved in primary care, while 44% offered emergency care, and 22% provided some form of prenatal care. Of those providers offering primary and emergency care, the largest share (100% in each case) were spending up to 30 hrs. per week at hospitality centers. Those providers contributing up to ten hours per week were mostly engaged in primary care (67%), while up to 33% were providing emergency and/or prenatal care.

Table 2 lists conditions presented by asylum seekers and refugees, ordered according to the highest proportion of providers who reported treating each illness. The most frequent condition treated was abdominal pain, with 25% of providers reporting this condition as highly common, and 75% as common. Dermatological conditions were the second most frequent condition with 14% of providers expressing this condition as highly common, almost 70% stating it as common, and 17% encountered this condition in some cases. Cardiac diseases and hypertension were considered common by 36% of providers, and 27% reported as observing these conditions in some cases. Headaches and neurological conditions were reported by 15% of providers as highly common, while 48% considered it a common condition; 36% did not observed it. Dehydration was treated by 54% of providers (9% highly common, and 45% common). Respiratory tract infection was considered by 36% of providers to be highly common, while 18% considered this condition common. Diabetes mellitus/metabolic was observed by 54% of providers, but only 18% of providers considered this condition highly common, 27% considered it a common issue, and 9% observed it in some cases. Injuries were reported as common by 32% of providers, while 23% saw injuries occasionally. Other conditions reported included psychiatric, dental, fever, and genitourinary issues. A total of 19 different conditions were identified including other types of infections, musculoskeletal pain, cancer and asthma. The category of other infection included mumps, varicella zoster virus, and sepsis.

**Table 2:** Frequency of conditions presented by asylum seekers and refugees as reported by volunteer providers (n = 75).

Condition	Percent of Physicians Reporting Each Condition			
	Highly Common	Common	Rare	Not Observed
Abdominal Pain	25%	75%	-	-
Dermatological	14%	69%	17%	-
Cardiac disease/Hypertension	-	36%	27%	36%
Headache/neurological	15%	48%	-	36%
Dehydration	9%	45%	-	45%
Respiratory tract infection	36%	18%	-	45%
Diabetes mellitus/metabolic	18%	27%	9%	45%
Injuries	-	32%	23%	45%
Psychiatric	-	27%	27%	45%
Dental	-	9%	36%	55%
Fever	27%	18%	-	55%
Genitourinary disease	-	27%	18%	55%
Allergic reactions	-	18%	27%	55%
Rheumatological	-	-	45%	55%
Gynaecological/obstetric	-	15%	30%	55%
Other Infections	-	-	27%	73%
Musculoskeletal pain	-	18%	-	82%
Cancer	-	-	18%	82%
Asthma	-	9%	-	91%

All volunteer providers reported dispensing analgesics, antipyretics, and prenatal vitamins as shown in Table 3. 86% of providers prescribed topical dermatological treatments, while 81% and 80% prescribed decongestants and allergy reliefs, and diabetes medications, respectively. 70% of providers reported dispensing antimicrobials, while 68% reported distributing gastrointestinal medications. Rehydration fluids were dispensed by 29% of providers, and 17% distributed antihypertensive and antiasthmatic medications.

**Table 3:** Types of medication dispensed to asylum seekers by participating volunteer providers (n = 75).

Medication Type	Percent of Providers Who Each	
	Prescribed	Did Not Prescribed
Analgesics /Antipyretics	100%	-
Prenatal Vitamins	100%	-
Topical Dermatological Treatments	86%	14%
Decongestants and Allergy Relief	81%	19%
Diabetes Medications	80%	20%
Antimicrobials	70%	30%
Gastrointestinal Medications	68%	32%
Rehydration fluids	29%	71%
Antihypertensive	17%	83%
Antiasthmatics	17%	83%

Table 4 presents a sensitivity analysis of estimated costs of treating the study population. The results reflect the use of different assumptions on reimbursement rates (CPT codes 99213 and CPT 99214), and number of patients per week as described during interviews with volunteer providers.

**Table 4:** Sensitivity analysis of estimated costs of treating asylum seekers and refugees entering El Paso, Texas October 2018 to February 2020 (costs estimates presented in 2020 US dollars).

Reimbursement Code	Cost Estimates Based on CMS Reimbursement Rates*					Costs Estimates Based on Private Insurers Rate Differences	
	October-2018 to January-2019	February to April, 2019		May-2019 to February-2020	Total Clinical Personnel *(a+b+c+d)*		
	Patients/week = 100 *(a)*	Patients/week = 250 *(b)*	Patients/week = 500 *(c)*	Patients/week = 50 *(d)*		135% *(e)*	180% *(f)*
*CPT—99213—Low to moderate severity problem (15 min)-Physicians*	$137,501	$427,187		$850,703	$1,415,391	$1,910,778	$2,547,704
			$694,003		$1,682,207	$2,270,979	$3,027,973
*CPT—99214—Moderate to high severity problem (25 min)-Physicians*	$202,561	$626,706		$1,235,406	$2,064,672	$2,787,308	$3,716,410
			$1,017,365		$2,455,332	$3,314,698	$4,419,598

*Note: reimbursement for medical students assumed as the standard volunteer cost per hour.

The difference between the two CPT codes was based in the estimated time spent with each patient varying between 15 to 25 minutes. From the period of October 2018 to January 2019, an approximate 100 patient were treated per week. This number varied between 250 to 500 patient per week during the February to April, 2019 period. Lastly, during the period of May 2019 to February 2020 the average number of patients treated per week was estimated at 50. The costs shown in columns *a—d*, Table 4, are estimates of total costs, reimbursements, for patient treatment. The last two columns (*e* and *f*) reflect estimates of total costs assuming two scenarios of higher reimbursement rates from private insurance of 135% and 180%, over the previously mentioned Medicaid reimbursement rates. Various sources confirm the differentials rates between government and private insurance reimbursement for the case of Medicare [30–33]. Total costs of providing care for asylum seekers and refugees were estimated to range between $1.9MM and $4.4MM. The highest costs were observed during the 3-month period of February-April 2019, which includes the period with the most number of arrivals per week. Given an estimated range on number of patients visits of 15,736 to 19,236 during the study period, cost per patient visit ranged between $99.33 and $229.76

## Discussion

This research presents cost estimates of providing care to refugees and asylum seekers entering the United States through El Paso, Texas, from Mexico during the period of October 2018 to February 2020. Clinicians volunteering at local non-for-profit hospitality centers provided primary, emergency and prenatal care services. This study also documented common conditions treated, and medications dispensed by these volunteer providers. The most common conditions included abdominal and dermatological issues, followed by cardiac, neurological, and dehydration. These issues were expected given the long journey covered by the study population. However, it was surprising that only 27% of providers considered psychiatric conditions common. Given the trauma faced by migrants both at their home country and throughout their travel, higher levels of mental health issues were expected. Since a large proportion of providers (44%) were involved in emergency care, and given the hectic pace of work in the hospitality centers, it is possible that issues like mental health were overlooked.

While the estimates of total costs of outpatient care presented here are significant ($1,9 MM to $4.4 MM), these are likely to be understated. These projections, for example, do not include costs of medications donated to hospitality centers or purchased by volunteer providers. The organizations involved did not keep records on medications, and a reliable estimate of costs was not possible. The assumptions of reimbursement (CPT-99213 and CPT-99214) refer to low, moderate, and high severity medical issues, which might not represent costs of emergency medical care provided by 44% of volunteer physicians as shown in Table 1. Similarly, the cost of transporting patients from hospitality center to hospital for admission were not included in this study; volunteers in private vehicles mostly did this activity and no records were kept. The analysis also did not cover costs of volunteer physician time spent on non-patient activities such as coordinating schedules, recruiting additional personnel, securing donations, and/or purchasing equipment needed at the hospitality centers during the peak months of migration. Lastly, the study did not present cost estimates of food and shelter needed during a patient’s recovery in a hospitality center before relocation. A realistic estimate of total costs is likely to be 200% ti 300% of the presented estimates.

The issues faced by El Paso County authorities and community organizations of providing care to migrants is not new. For several generations, Border communities have faced the need to support waves of new arrivals. As the numbers of migrant reaching the US Border are likely to continue and intensify, relying on volunteer providers, community organizations, and poorly funded local governments alone to provide care to refugees and asylum seekers is an unsustainable approach. Comments by volunteer providers, for example, reflect the difficult conditions under which they had to operate during the study period, and offer general indications on the pressure placed on local hospitality and healthcare resources. Rather, a well-planned and financed strategy leveraging local community and government resources is likely to have a better, long-lasting impact. Further, there is a clear need for Federal government support, which in the past has been insufficient for El Paso County. For example, the $2.9MM received by El Paso in 2019 [34], was lower than the high estimate of $4.4MM in this study.

An important area that merits further research is the development of mechanisms for closer coordination of care between institutions in both sides of the US-Mexico Border. This could include investment on existing border public health organizations, and the implementation of technology-based solutions. The use of technology for improved coordination between local organizations, the Immigration and Customs Enforcement (ICE), US Customs and Border Protection, and the Office of Refugee Resettlement is critical for improved care for migrants. For example, upon detention at the Border refugees suffering with diabetes had to surrender medication such as insulin. This requirement, while necessary for controlling import of illegal drugs likely resulted in extreme harm for certain migrant groups; a technology-based system for care coordination is likely to prevent unnecessary harm to migrants requiring essential medication. Lastly, it is important to identify appropriate mechanisms to finance healthcare services for migrating populations, particularly in cases where several countries are involved. One venue could be the implementation of cross-national insurance programs designed to support communities sharing international boundaries. Such insurance programs would allow these communities to receive timely and appropriate funding to provide healthcare services and other forms of humanitarian aid to displaced populations. The case of El Paso and Juarez, as well as similar cases of communities in Europe, Asia and Africa, reflect the need for risk-mitigation funding mechanisms in place, which could be offered by international development organizations such as the World Bank. Further, these programs would alleviate differences in the kind of support displaced populations receive given resource disparities, or political will differences that could exist between different countries.

While the solution to the problems of large waves of refugees and asylum seekers will require significant political and economic changes in home countries, it is clear that such changes will take many years. In the meantime, it is important to find solutions to dealing with health crises faced by individuals arriving at our borders. It is our hope that the results and points covered in this study will contribute to finding such solutions.

## Conclusion

This study provided estimates of the cost of care provided by volunteer clinicians to refugees and asylum seekers entering the United States through El Paso, Texas, from Mexico between October 2018 to February 2020, a period of unprecedented large migrations. This is the first study looking at the cost of healthcare for refugees and asylum seekers provided by volunteer clinicians, in a binational context. The result from this study should help provide the basis for the development of sustainable policies for improving the health of refugee and asylum seeker populations.

## Supporting information

S1 Table (PDF)

## References

[1] U.S.C. and B.P. US Department of Homeland Security, Southwest Border Migration FY 2019 |, Southwest Bord. Migr. FY 2019Y 2019. (2019).

[2] Houston Chronicle, (n.d.). http://digital.olivesoftware.com/Olive/ODN/houstonchronicle/Default.aspx (accessed November 1, 2019).

[3] Price S., Borders Without Enough Doctors: El Paso Physicians Volunteer Care for Asylum Seekers, TMA Publ. (2019). 30995335

[4] Volunteer Doctors At The U.S.-Mexico Border Face Overwhelming Need: Shots—Health News: NPR, (n.d.). https://www.npr.org/sections/health-shots/2019/04/05/709965103/makeshift-volunteer-clinics-struggle-to-meet-medical-needs-at-the-border (accessed January 30, 2020).

[5] M.D.S. and Z. Kanno-Youngs, Biden Issues Orders to Dismantle Trumpʼs ʻAmerica Firstʼ Immigration Agenda, New York Times. (2021).

[6] M.J. and M. Rivlin-Nadler, Migrant Families Force Biden to Confront New Border Crisis,—New York Times. (2021).

[7] H. M. and Lara Jakes M.D.S., Kanno-Youngs Zolan, Biden and Immigration: President to Welcome More Refugees, but Far From All Will Get In—The New York Times, New York Times. (2021).

[8] Jordan Miriam, Thousands of Migrant Children Detained in Resumption of Trump-Era Policies, New. (2021).

[9] R. Luscombe, Last Haitian migrants removed from Del Rio encampment, homeland security chief says | US immigration | The Guardian, Guard. (n.d.). https://www.theguardian.com/us-news/2021/sep/24/del-rio-encampment-immigration-texas (accessed December 21, 2021).

[10] Gulacti U., Lok U., Polat H., Emergency department visits of Syrian refugees and the cost of their healthcare., Pathog. Glob. Health. 111 (2017) 219–224. doi: 10.1080/20477724.2017.1349061 28720037 PMC5560198

[11] Requena-Méndez A., Bussion S., Aldasoro E., Jackson Y., Angheben A., Moore D., et al, Cost-effectiveness of Chagas disease screening in Latin American migrants at primary health-care centres in Europe: a Markov model analysis, Lancet Glob. Heal. 5 (2017) e439–e447. doi: 10.1016/S2214-109X(17)30073-6 28256340

[12] Imaz-Iglesia I., Miguel L.G.S., Ayala-Morillas L.E., García-Pérez L., González-Enríquez J., Blasco-Hernández T., et al, Economic evaluation of chagas disease screening in Spain, Acta Trop. 148 (2015) 77–88. doi: 10.1016/j.actatropica.2015.04.014 25917718

[13] Sicuri E., Muñoz J., Pinazo M.J., Posada E., Sanchez J., Alonso P.L., et al, Economic evaluation of Chagas disease screening of pregnant Latin American women and of their infants in a non endemic area., Acta Trop. 118 (2011) 110–7. doi: 10.1016/j.actatropica.2011.02.012 21396345

[14] Goetz K., Winkelmann W., Steinhäuser J., Assessment of oral health and cost of care for a group of refugees in Germany: A cross-sectional study, BMC Oral Health. 18 (2018) 69. doi: 10.1186/s12903-018-0535-1 29699553 PMC5921436

[15] Ortega L., Meltzer M., Weinberg M., Liu Y., Armstrong G., Messonier M., Cost of Vaccinating Refugees Overseas Versus After Arrival in the United States, 2005, CDC MMWR Wkly. 57 (2008) 229–232. 18322445

[16] Joo H., Maskery B., Mitchell T., Leidner A., Klosovsky A., Weinberg M., A comparative cost analysis of the Vaccination Program for US-bound Refugees, Vaccine. 36 (2018) 2896–2901. doi: 10.1016/j.vaccine.2017.09.023 28919225 PMC5851828

[17] Pezzi C., McCulloch A., Joo H., Cochran J., Smock L., Frerich E., et al, Vaccine delivery to newly arrived refugees and estimated costs in selected U.S. clinics, 2015, Vaccine. 36 (2018) 2902–2909. doi: 10.1016/j.vaccine.2017.12.023 29395535 PMC6961801

[18] Adachi K., Coleman M.S., de la Motte Hurst C., Vargas M.L., Oladele A., Weinberg M.S., Costs of, and reimbursement for, vaccines: A case study at the Board of Health Refugee Services in DeKalb county, Georgia, Vaccine. 31 (2013) 2317–2322. doi: 10.1016/j.vaccine.2012.08.016 22910285

[19] Muenning P., Pallin D., Sell R., Chan M.-S., The Cost Effectiveness of Strategies for the Treatment of Intestinal Parasites in Immigrants, N. Engl. J. Med. 340 (1999) 773–779. doi: 10.1056/NEJM199903113401006 10072413

[20] Zammarchi L., et.al, A scoping review of cost-effectiveness of screening and treatment for latent tuberculosis infection in migrants from high-incidence countries, BMC Health Serv. Res. 15 (2015). doi: 10.1186/s12913-015-1045-3 26399233 PMC4581517

[21] Coleman M.S., Garbat-Welch L., Burke H., Weinberg M., Humbaugh K., Tindall A., Cambron J., Direct costs of a single case of refugee-imported measles in Kentucky, Vaccine. 30 (2012) 317–321. doi: 10.1016/j.vaccine.2011.10.091 22085555

[22] DuBard C.A., Massing M.W., Trends in Emergency Medicaid Expenditures for Recent and Undocumented Immigrants, JAMA. 297 (2007) 1085–1092. doi: 10.1001/jama.297.10.1085 17356029

[23] Goldman D.P., Smith J.P., Sood N., Immigrants and the Cost of Medical Care, Health Aff. 25 (2006) 1700–1711. doi: 10.1377/hlthaff.25.6.1700 17102196

[24] Mohanty S.A., Woolhandler S., Himmelstein D.U., Pati S., Carrasquillo O., Bor D.H., Health care expenditures of immigrants in the United States: A nationally representative analysis, Am. J. Public Health. 95 (2005) 1431–1438. doi: 10.2105/AJPH.2004.044602 16043671 PMC1449377

[25] Independent Sector, Value of Volunteer Time/ State and Historical Data, (2020).

[26] U.S.B. of L. Statistics, Consumer Price Index, (2021). https://www.bls.gov/cpi/ (accessed December 21, 2021).

[27] Drummond M.F., O’Brien B., Stoddart G.L., Torrance G.W., Methods for the Economic Evaluation of Health Care Programmes, Second Edition, Am. J. Prev. Med. 14 (2005) 243. doi: 10.1016/S0749-3797(97)00069-X

[28] Sanders G.D., Neumann P.J., Basu A., Brock D.W., Feeny D., Krahn M., et al, Recommendations for conduct, methodological practices, and reporting of cost-effectiveness analyses: Second panel on cost-effectiveness in health and medicine, JAMA—J. Am. Med. Assoc. 316 (2016) 1093–1103. doi: 10.1001/jama.2016.12195 27623463

[29] Eisman A.B., Kilbourne A.M., Dopp A.R., Saldana L., Eisenberg D., Economic evaluation in implementation science: Making the business case for implementation strategies, Psychiatry Res. 283 (2020). doi: 10.1016/j.psychres.2019.06.008 31202612 PMC6898762

[30] Biener A.I., Selden T.M., Public and private payments for physician office visits, Health Aff. 36 (2017) 2160–2164. doi: 10.1377/hlthaff.2017.0749 29200346

[31] Selden T.M., Karaca Z., Keenan P., White C., Kronick R., The growing difference between public and private payment rates for inpatient hospital care, Health Aff. 34 (2015) 2147–2150. doi: 10.1377/hlthaff.2015.0706 26643636

[32] Selden T.M., Differences between public and private hospital payment rates narrowed, 2012–16, Health Aff. 39 (2020) 94–99. doi: 10.1377/hlthaff.2019.00415 31905058

[33] Wallace J., Song Z., Traditional medicare versus private insurance: How spending, volume, and price change at age sixty-five, Health Aff. 35 (2016) 864–872. doi: 10.1377/hlthaff.2015.1195 27140993 PMC4943661

[34] Dearman E., Texas receives $2.9 million in reimbursement for migrant care, El Paso Times. (2019). https://www.elpasotimes.com/story/news/politics/2019/10/11/texas-receives-2-9-million-reimbursement-migrant-care/3944458002/ (accessed December 21, 2021).

