# Supplementary material for: Cost of care for asylum seekers and refugees entering the United States: The case of volunteer medical providers in El Paso, Texas
Source: PLoS One. 2022 Dec 1;17(12):e0278386. doi: 10.1371/journal.pone.0278386 (PMC9714800; doi:10.1371/journal.pone.0278386)
Supplement: S1 Table — (PDF) [file pone.0278386.s001.pdf]

DETAIL OF COST ESTIMATES FOR THE PERIOD: JUNE 2019 TO FEBRUARY 2020

Nominal Reimbursements	***2019***	***2020***
CPT - 99213 - Low to moderate severity problem (15 min)-Physicians	$75.32	$76.15
CPT - 99214 - Moderate to high severity problem (25 min)-Physicians	$110.28	$110.43

inflation rate			
	**2019**	**255.7**	**1.80%**
	**2020**	**258.8**	**1.20%**

Inflation Adjusted Reimbursements	***2020***	***2020***
CPT - 99213 - Low to moderate severity problem (15 min)-Physicians	$76.23	$76.15
CPT - 99214 - Moderate to high severity problem (25 min)-Physicians	$111.62	$110.43

CPT 99213 Reimbursements

**Health Professional Volunteers (Hrs)**	**Period: June 27, 2019 to December 31, 2019**					
	**Number of Volunteer Hours***	**Assumed Reimbursement (CMS rate per visit)**	**Assumed time spent per patient (mins)**	**Assumed number of patients treated per hr**	**Assumed number of patients treated**	**Total Calculated Cost**
Licensed Physicians	1,946	$76.23	15	4	7,784	$593,399
Licensed Physician Assistant	48	$76.23	15	4	192	$14,637
License Nurse Practitioner	74	$76.23	15	4	296	$22,565
RNs	128	$25.43	15	4	510	$12,969
Medical Students**	15	$25.43	15	4	60	$1,526
Total	2,211					$645,096

*Source: United Way of El Paso

**Note: reimbursement for medical students was assumed as the standard volunteer cost per hour

CPT 99214 Reimbursements

Health Professional Volunteers (Hrs)	Period: June 27, 2019 to December 31, 2019					
	Number of Volunteer Hours*	Assumed Reimbursement (CMS rate per visit)	Assumed time spent per patient (mins)	Assumed number of patients treated per hr	Assumed number of patients treated	Total Calculated Cost
Licensed Physicians	1,946	$111.62	15	4	7,784	$868,827
Licensed Physician Assistant	48	$111.62	15	4	192	$21,430
License Nurse Practitioner	74	$111.62	15	4	296	$33,039
RNs	128	$25.43	15	4	510	$12,969
Medical Students**	15	$25.43	15	4	60	$1,526
Total	2,211					$937,791

*Source: United Way of El Paso

**Note: reimbursement for medical students was assumed as the standard volunteer cost per hour

DETAIL OF COST ESTIMATES FOR THE PE

Nominal Reimbursements
CPT - 99213 - Low to moderate severity problem (15 min)-Physicians
CPT - 99214 - Moderate to high severity problem (25 min)-Physicians

Inflation Adjusted Reimbursements
CPT - 99213 - Low to moderate severity problem (15 min)-Physicians
CPT - 99214 - Moderate to high severity problem (25 min)-Physicians

CPT 99213 Reimbursements

Health Professional Volunteers (Hrs)	Period: January 1, 2020 to February 28, 2020						Overall Total Cost
	Number of Volunteer Hours*	Assumed Reimbursement (CMS rate per visit)	Assumed time spent per patient (mins)	Assumed number of patients treated per hr	Assumed number of patients treated	Total Calculated Cost	
Licensed Physicians	663	$76.15	15	4	2,652	$201,950	$795,349
Licensed Physician Assistant	8	$76.15	15	4	32	$2,437	$17,074
License Nurse Practitioner	0	$76.15	15	4	0	$0	$22,565
RNs	12	$25.43	15	4	48	$1,221	$14,190
Medical Students**	0	$25.43	15	4	0	$0	$1,526
Total	683					$205,607	$850,703

*Source: United Way of El Paso

**Note: reimbursement for medical students was ass

CPT 99214 Reimbursements

Health Professional Volunteers (Hrs)	Period: January 1, 2020 to February 28, 2020						Overall Total Cost
	Number of Volunteer Hours*	Assumed Reimbursement (CMS rate per visit)	Assumed time spent per patient (mins)	Assumed number of patients treated per hr	Assumed number of patients treated	Total Calculated Cost	
Licensed Physicians	663	$110.43	15	4	2,652	$292,860	$1,161,687
Licensed Physician Assistant	8	$110.43	15	4	32	$3,534	$24,964
License Nurse Practitioner	0	$110.43	15	4	0	$0	$33,039
RNs	12	$25.43	15	4	48	$1,221	$14,190
Medical Students**	0	$25.43	15	4	0	$0	$1,526
Total	683					$297,615	$1,235,406

*Source: United Way of El Paso

**Note: reimbursement for medical students was ass

DETAIL OF COST ESTIMATES FOR THE PERIOD: OCTOBER 2018 TO APRIL 2019

Nominal Reimbursements	***2018***	***2019***
CPT - 99213 - Low to moderate severity problem (15 min)-Physicians	$74.16	$75.32
CPT - 99214 - Moderate to high severity problem (25 min)-Physicians	$109.44	$110.28

Inflation Rate (2018-2019)

inflation adjusted	***2020***	***2020***
CPT - 99213 - Low to moderate severity problem (15 min)-Physicians	$76.43	$76.23
CPT - 99214 - Moderate to high severity problem (25 min)-Physicians	$112.80	$111.62

Scenario 1: 250 patients per day -

**Health Professional Type**	**Period: October 1, 2018 to Jan 31, 2019**	**Period: February 1, 2019 to April 30, 2019**
Number of patients treated	1,800	3,500
Number of volunteers hours		
Licensed Physicians	375	810.2
Licensed Physician Assistant	0	0.0
Licensed Nurse Practitioner	75	64.8
RNs	0	0.0
Medical Students	0	0.0
**Estimated Total Cost**		
CPT - 99213 - Low to moderate severity problem (15 min)	$76.23	$76.23
CPT - 99214 - Moderate to high severity problem (25 min)	$111.62	$111.62
Total Cost (CPT 99213)	$137,501	$427,187
Total Cost (CPT 99214)	$202,561	$626,706

Scenario 2: 500 patients per day -

**Health Professional Type**	**Period: October 1, 2018 to Jan 31, 2019**	**Period: February 1, 2019 to April 30, 2019**
Number of patients treated	1,800	7,000
Number of volunteers hours		
Licensed Physicians	375	810.2
Licensed Physician Assistant	0	0.0
Licensed Nurse Practitioner	75	64.8
RNs	0	0.0
Medical Students	0	0.0
**Estimated Total Cost**		
CPT - 99213 - Low to moderate severity problem (15 min)	$76.23	$76.23
CPT - 99214 - Moderate to high severity problem (25 min)	$111.62	$111.62
Total Cost (CPT 99213)	$137,501	$694,003
Total Cost (CPT 99214)	$202,561	$1,017,365
